# Effectiveness of a Program for Pain Intensity and Knee Osteoarthritis Reduction Among Elderly Individuals in Thailand: A Randomized Controlled Trial Study

**DOI:** 10.1002/iid3.70604

**Published:** 2026-11-13

**Authors:** Pakorn Pusuwan, Phanit Srisuttha, Raweephun Sunanta, Keng Chaichana, Nitikorn Phoosuwan

**Affiliations:** ^1^ Department of Applied Thai Traditional Medicine University of Phayao Phayao Thailand; ^2^ Faculty of Public Health Thammasat University Pathum Thani Thailand; ^3^ Department of Public Health and Caring Sciences, Faculty of Medicine Uppsala University Uppsala Sweden

**Keywords:** elderly people, Thai, traditional medicine, treatment, visual analog scale

## Abstract

**Aim:**

The objective of the study was to assess the effects of the Pain Intensity and Knee Osteoarthritis Reduction (PIKOR) program on pain and severity of knee osteoarthritis (KOA) among older people in Thailand.

**Methods:**

This randomized controlled trial, conducted in Northern Thailand, enrolled 58 older adults with KOA. Participants were randomly allocated to either an intervention group, which received the PIKOR program, or a control group receiving standard care. The intervention consisted of the PIKOR program, which combined Court‐Type Traditional Thai Massage (CTTM), Hot Herbal Compress (HHC), Leg and Knee Stretching (LKS), and Herbal Knee Poultice (KPH). This integrated protocol sought to maintain therapeutic efficacy while minimizing the direct time burden on the Thai traditional physician. Treatment outcomes were evaluated using the Visual Analog Scale (VAS) and the Western Ontario and McMaster Universities Osteoarthritis Index (WOMAC). Statistical significance was determined at a 95% confidence level (*p* < 0.05), utilizing both independent and paired *t*‐tests for inter‐group and intra‐group comparisons, respectively.

**Results:**

Participants in the intervention group demonstrated greater reductions in mean VAS and WOMAC scores compared with those in the control group.

**Conclusion:**

The PIKOR program resulted in significantly greater reductions in pain, stiffness, and functional limitations among older adults in the intervention group compared with those in the control group. It may represent a viable alternative for managing KOA, particularly by reducing the time required for consultation with a Thai traditional medicine practitioner.

**Trial Registration:** Thai Clinical Trial Registry: TCTR20260415004 on April 15, 2026 (https://www.thaiclinicaltrials.org/export/pdf/TCTR20260415004).

## 1 Introduction

Osteoarthritis is a degenerative joint disorder characterized by the progressive deterioration of articular cartilage, accompanied by structural changes in surrounding joint tissues, which collectively result in pain, swelling, and joint stiffness. It most commonly affects the knees, hips, spine, and hands [1]. Knee osteoarthritis (KOA) represents the most common form of arthritis among older adults and is a major contributor to disability worldwide [2]. There were about 365 million KOA sufferers, 29.51 million new cases (incidence), and 11.53 million disability‐adjusted life years in 2019 [3]. Knee osteoarthritis (KOA) has an estimated prevalence of 22.9% among individuals aged ≥ 40 years and 19.2% within Asian populations [4], with approximately 60%–90% of adults aged ≥ 65 years affected [5]. Women are disproportionately affected, experiencing both higher prevalence rates and more severe symptoms than men [5].

Thailand, an upper‐middle‐income nation, is seeing a demographic shift toward an ageing society, with 20% of the population presently being 60 years of age or older [6]. As a result, KOA has become increasingly prevalent. It represents a significant public health concern [7, 8]. Older adults face an estimated 80%–90% risk of developing the condition, affecting over 6 million individuals and making it one of the leading causes of disability among older people [7], particularly in women, with prevalence increasing with age [8]. KOA treatment in Thailand includes pharmacological therapy, non‐pharmacological therapy, and surgical intervention for severe cases [8, 9]. Conventional therapies of KOA for preventing pain include: dietary, resting, exercise, medicines, laser therapy [10], and toxin injection therapy [11]. Integrative medicines, like Hot Herbal Compression (HHC), Court‐Type Thai Traditional Massage (CTTM), and Herbal Knee Poultice [9] from Thai traditional medicine play a growing role in managing chronic diseases, especially those involving the bones, joints, and muscles [9]. Thai traditional medicine can be integrated with modern medical treatment, especially for patients who cannot take pain‐relieving medications or experience adverse effects from non‐steroidal anti‐inflammatory drugs (NSAIDs), such as those with kidney disease or gastritis [9]. Interestingly, clinical presentations of osteoarthritis do not differ between rural and urban Thai populations, although urban patients tend to have greater access to treatment options [8]. Despite the significant impact of Thai traditional medicine on KOA in Thailand, research on the effectiveness of traditional Thai medicine as a treatment program for KOA in older adults remains limited. Therefore, this study aimed to evaluate a program designed to reduce pain and improve outcomes in individuals with knee osteoarthritis. The program may serve as a potential alternative approach for alleviating pain and managing KOA among older adults.

## 2 Methods

### 2.1 Aim

This randomized controlled trial evaluated the effects of the Pain Intensity and Knee Osteoarthritis Reduction (PIKOR) program among older adults in Thailand.

### 2.2 Study Design and Setting

The study was carried out in the northern Thai province of Phayao, which is roughly 600 km away from Bangkok. This province is characterized by its diverse geographical landscape and cultural heritage. Notably, Phayao has a significant number of older adults, around 100,000, representing more than 20% of the province's population [12].

### 2.3 Participants

Participants were recruited from a district in Phayao, which was identified as having the highest proportion of older adults [12]. The sample size was calculated using G*Power statistical software, based on an assumed effect size of 0.8, statistical power of 80% (1 − *β*), a significance level (*α*) of 0.05, and an allocation ratio of 1:1 between the intervention and control groups. An extra 10% was included in the calculated sample size to account for potential attrition during the follow‐up period, requiring a minimum of 26 participants in each group. Inclusion criteria were people who: (1) aged 60–79 years old, (2) reported pain in one knee for at least 6 months, (3) had no history of knee replacement surgery, and (4) provided informed consent to participate in the study. Exclusion criteria included a fracture or injury involving the knee joint, a diagnosis of any cancer, any form of paralysis, or current use of NSAIDs.

### 2.4 Procedure

The study received ethical approval from the University of Phayao Human Ethics Committee prior to participant recruitment (COA: UP‐HEC 1.3/040/65). Thereafter, authorization was granted by the heads of the District Public Health Office to carry out the data collection process. The researchers received a list of individuals residing in the selected sub‐district, which was used to identify and screen for eligibility. Eligible participants were invited to take part in the study. An invitation covering a socio‐demographic questionnaire (gathering information on age, gender, marital status, and education level), and a detailed information sheet about the study was sent from the researchers (P.P. and P.S.) to the eligible participants. Fifty‐eight older adults agreed to participate and signed an informed consent form. Participants were randomly allocated to either the intervention or control group using a sequentially numbered block randomization method, conducted by the researchers (P.P and N.P.). Participants in the intervention group received the PIKOR program, whereas those in the control group were provided with routine care (i.e., CTTM for 45 min combined with HHC for 15 min administered three times within 1 week) from the Sub‐district Health Promoting Hospital (SHPH) (Figure 1). The study took place between October 1, 2022 and February 28, 2023. The trial was retrospectively registered with the Thai Clinical Trial Registry because prospective trial registration was not mandated by local institutional requirements. There was no change to the trial after the study started.

**Figure 1:**
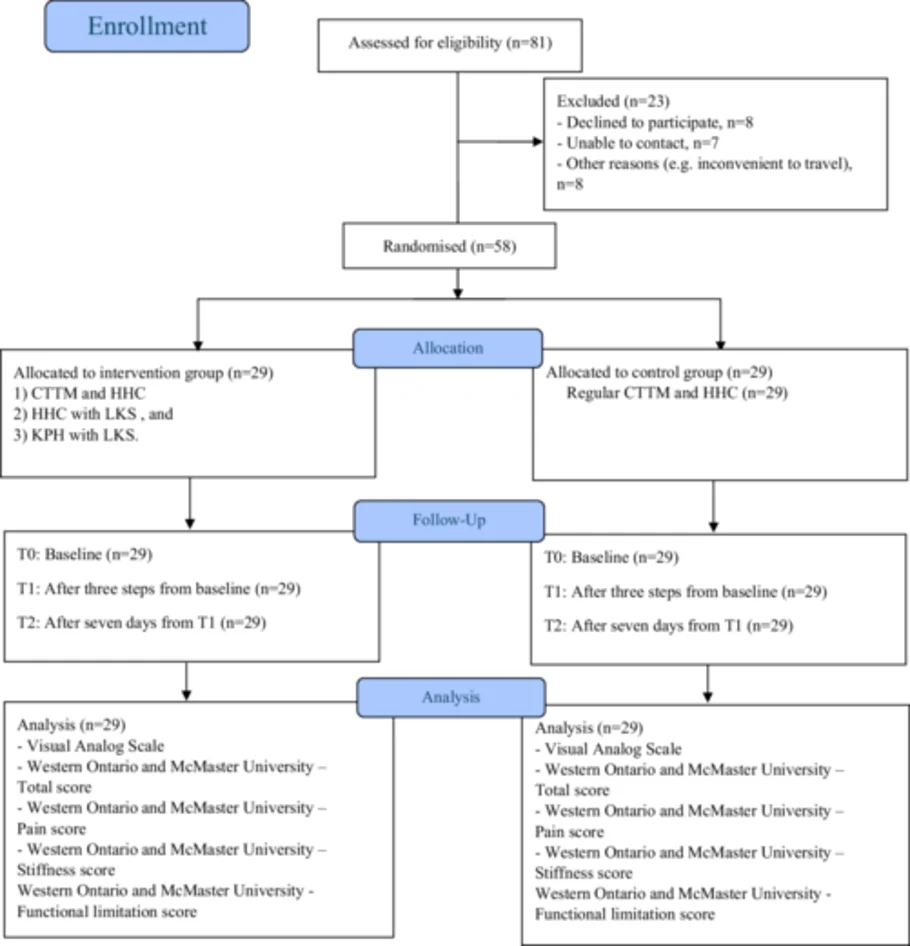
Flow diagram of the study.

### 2.5 The Pain Intensity and Knee Osteoarthritis Reduction (PIKOR) Program

The researchers developed the PIKOR program as a traditional Thai medicine intervention to reduce knee osteoarthritis pain in older adults. The PIKOR program was delivered by a licensed Applied Thai Traditional Medical Practitioner (TTMP) and comprised three sequential components: step (1) Court‐Type Traditional Thai Massage (CTTM) combined with Hot Herbal Compression (HHC); step (2) HHC in conjunction with Legs and Knee Stretching (LKS); and step (3) Knee Poultice by Herbs (KPH) combined with LKS. Each step was separated by a 2‐day interval. Outcome assessment was conducted on the seventh day after completion of the final intervention session by P.P. and K.C. Participant identification codes were used during follow‐up assessments, and the outcome assessors were blinded to group allocation throughout the evaluation process.

Step 1: CTTM combined with HHC. CTTM is a traditional Thai massage technique comprising five sequential procedures: (1) application of pressure along the four‐line leg pathways, with focused compression of the femoral artery to enhance blood circulation to the toes; (2) targeted pressure on three specific points surrounding the knee (designated as points 1–3); (3) massage of four designated points along the outer leg, with particular emphasis on the fourth point; (4) mobilization of the knee joint combined with pressure applied to the popliteal region; and (5) massage of four specific points along the inner leg, emphasizing the third and fourth points, followed by knee joint mobilization (Figure 2). Following the CTTM, HHC was applied to the legs and knees for 15 min, using the herbal compresses that contain a blend of Thai medicinal herbs to reduce inflammation and relieve pain. The total duration of this step was 60 min (Figure 3).

**Figure 2:**
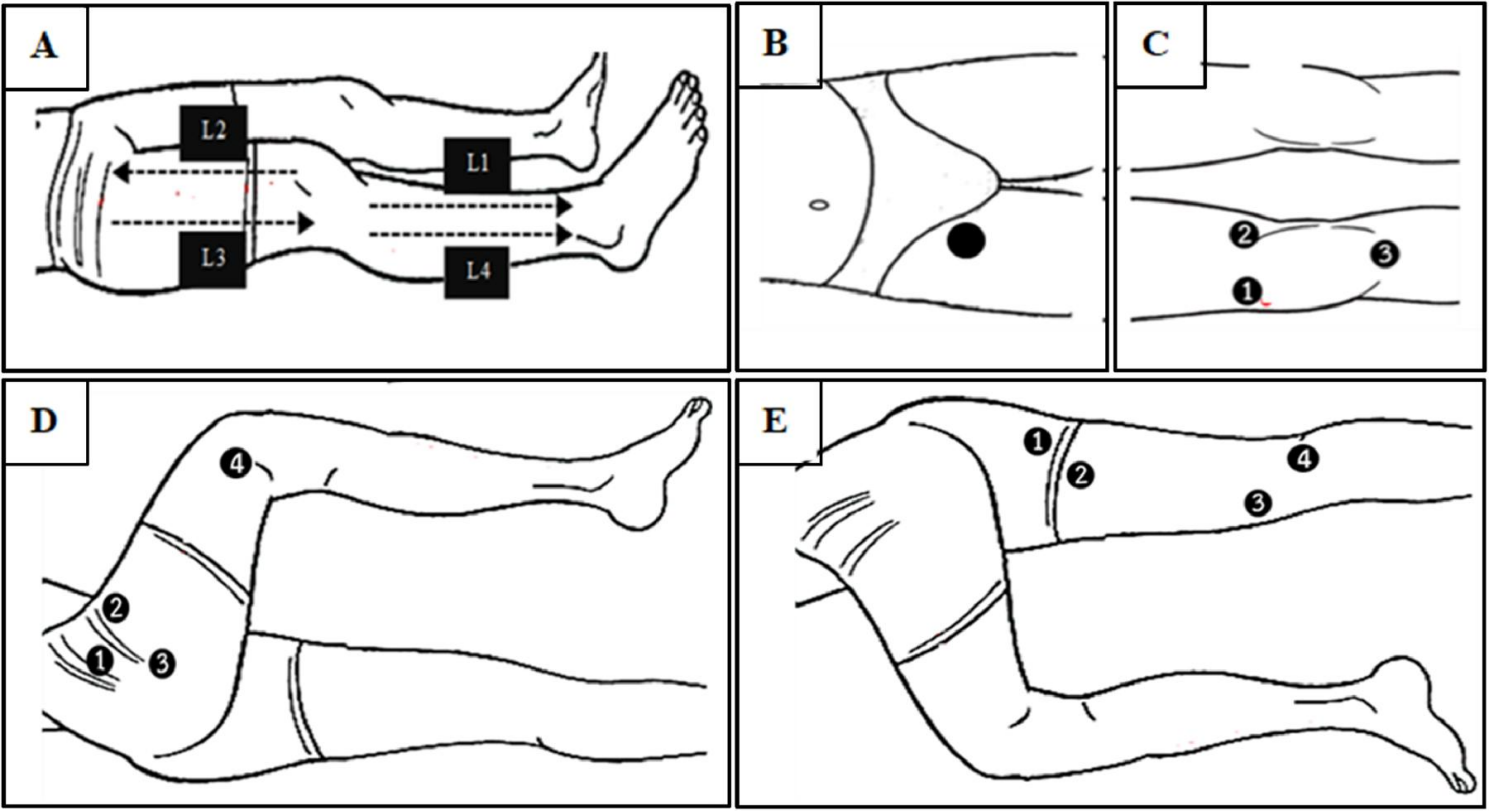
(A) Lines on legs L1–L4. (B) Femoral artery point. (C) Three points around the knee. (D) Four points on the outer leg. (E) Four points on inner leg.

**Figure 3:**
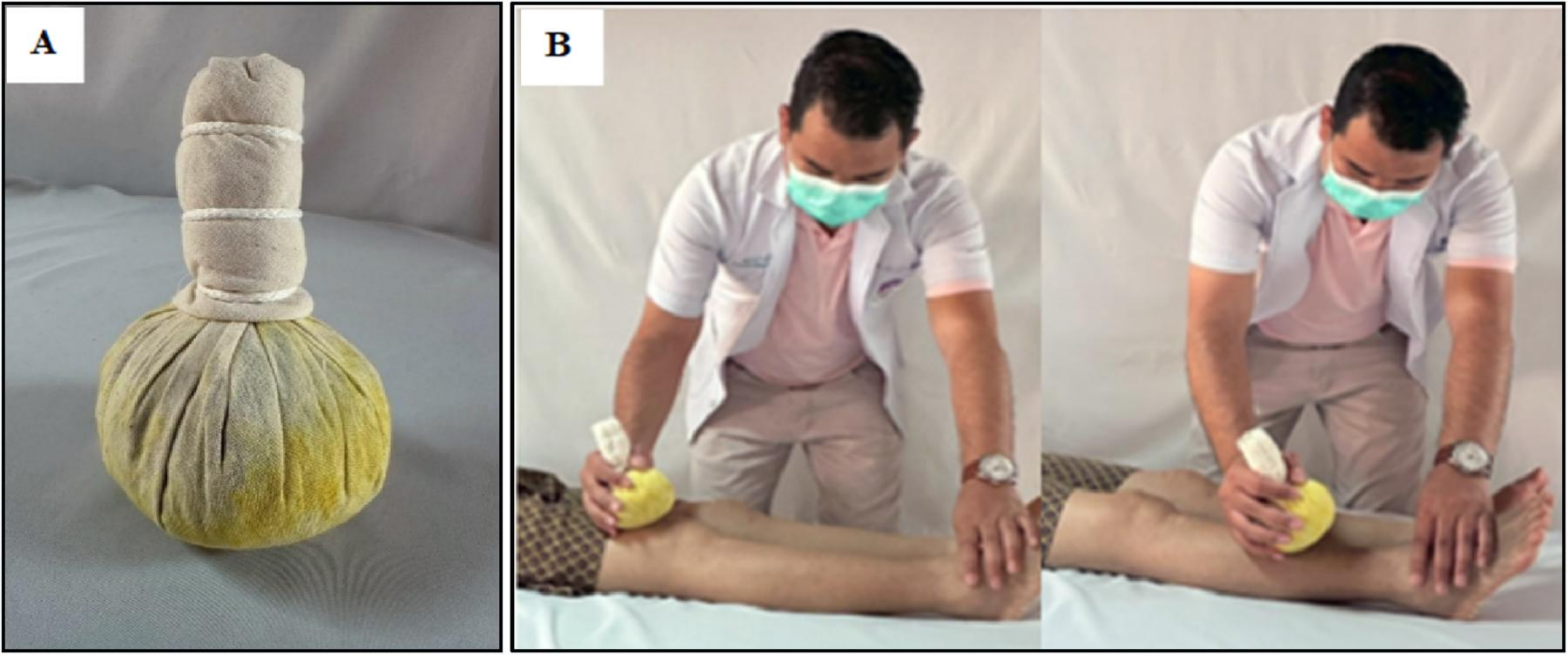
(A) Luk pra kob. (B) Hot herbal compression on knee joints and leg.

Step 2: HHC combined with LKS. This step began with the administration of HHC, followed by LKS. The LKS was developed to improve joint mobility by stretching the leg and knee musculature. This phase comprised two exercise postures: (1) for the quadriceps exercise, participants were positioned either supine or seated with both legs extended, raised their toes, and performed an isometric contraction of the quadriceps muscles, holding the contraction for 10 s before alternating to the contralateral leg. This was repeated alternately for a total of 10 min. (2) Tiptoe exercise: participants began standing with their feet apart and hands placed against a wall. While standing on one tiptoe, they bent the opposite leg upward in a natural position, contracted the leg and knee muscles, and held the position for 10 s before switching sides. This posture was repeated over 10 min (Figure 4). The total duration was 35 min.

**Figure 4:**
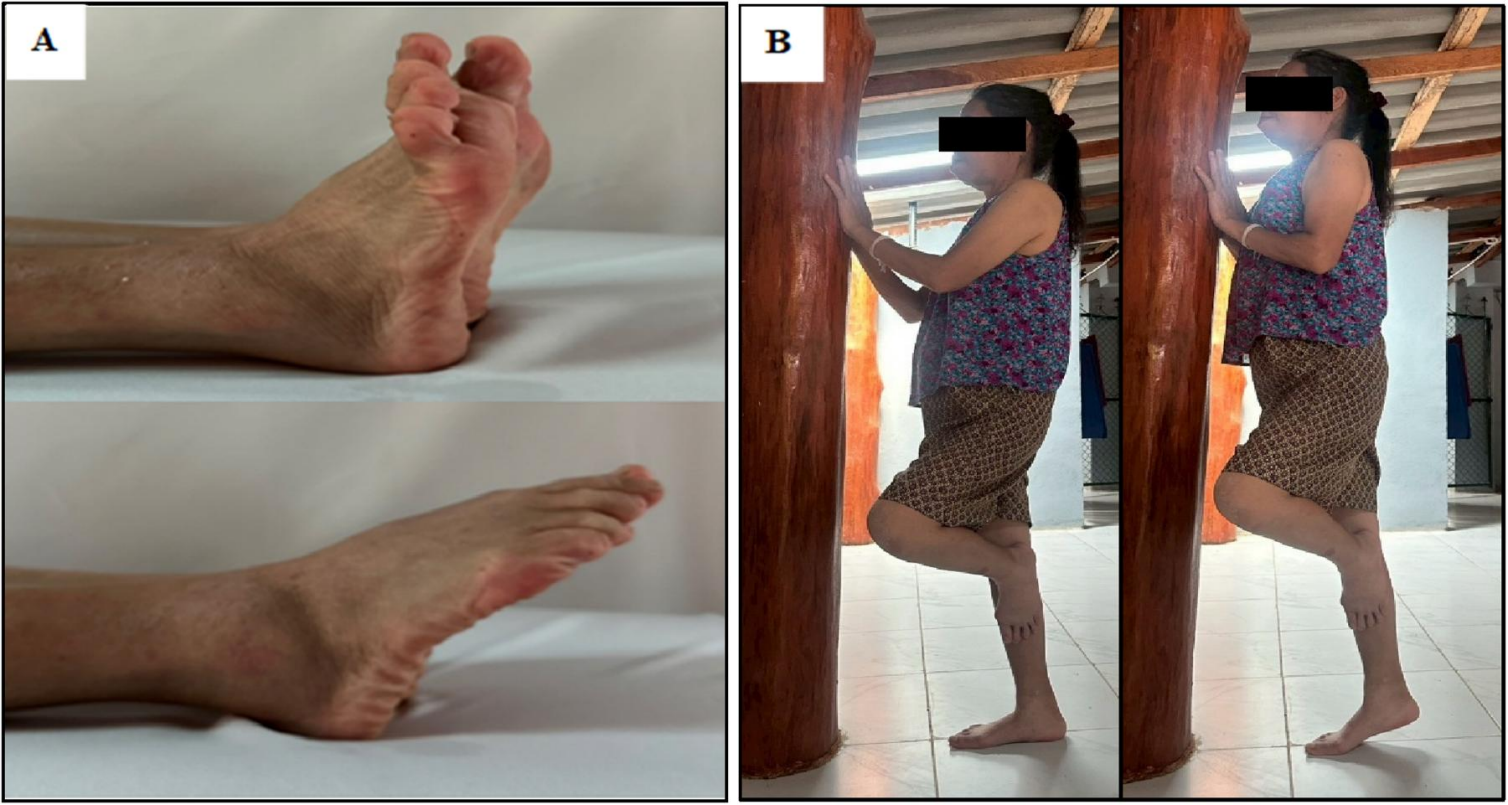
Legs and knee stretching: (A) quadriceps exercise. (B) Tiptoe exercise.

Step 3: KPH combined with LKS. This step began with KPH and was followed by LKS. The KPH consisted of applying herbal poultice formulated from a powdered blend of *Gloriosa superba* L. and *Zingiber officinale* Roscoe (ginger) to the knee region for 15 min (Figure 5). Afterwards, LKS was applied for 20 min. The total duration of Step 3 was 35 min.

**Figure 5:**
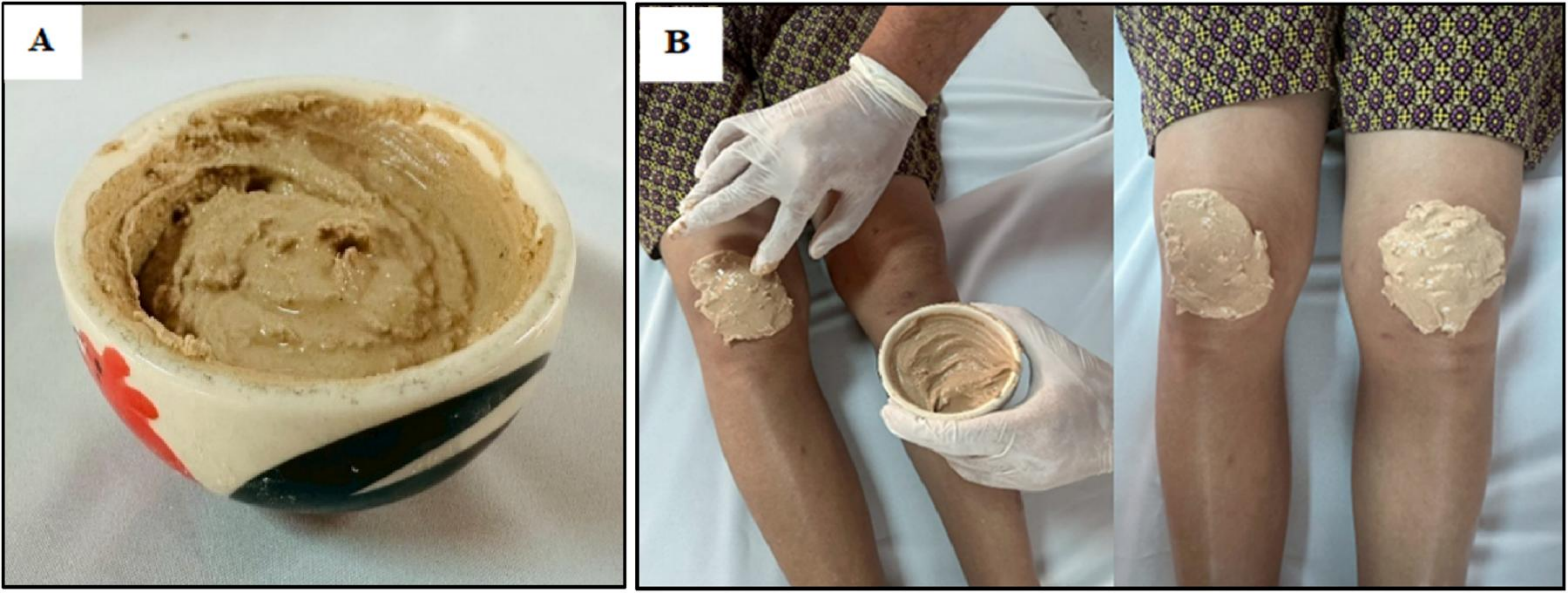
(A) Knee poultice by herbs. (B) Applied herbal mask on knee area.

### 2.6 Instruments

The researchers developed a questionnaire specifically for this study, comprising three sections: (1) sociodemographic characteristics of the participants; (2) pain score assessment; and (3) severity of knee osteoarthritis. The content validity of the questionnaire was evaluated by three experts using the Index of Item‐Objective Congruence (IOC), with IOC scores ranging from 0.6 to 1.0. The questionnaire was pretested for reliability among older adults (*n* = 30) in another province, where a Cronbach's alpha coefficient = 0.95.

The sociodemographic characteristics included specific information about the participants, for example, gender, age, body mass index, and physical activity.

Pain score was assessed using the VAS [13], a widely recognized and frequently used tool for evaluating pain intensity. The VAS is considered a reliable, valid, and responsive measure. It consists of a 10‐cm horizontal line with two labels: “no pain” (0) and “worst possible pain” (10). Participants were instructed to indicate their current pain level by placing a vertical mark along the line, corresponding to the intensity of pain they were experiencing.

The pain, stiffness, and functional limitations of knee osteoarthritis were evaluated using the Thai version of the Western Ontario and McMaster University (WOMAC) index, which contains 22 items, modified by Kuptniratsaikul and Rattanachaiyanont [14], with a Cronbach's alpha coefficient ranging from 0.65 to 0.71. The WOMAC is organized into three dimensions: pain (5 items), stiffness (2 items), and function (15 items). Each item was scored on a scale from 0 to 10 using a 10‐cm horizontal line with a numeric description, where 0 indicates “no symptoms” and 10 indicates “extreme symptoms”. The total possible score on the WOMAC ranged from 0 to 220, with a higher score indicating worse pain, stiffness, and poorer physical function. The pain level was evaluated using questions 1–5, with a total possible score of the pain level ranging between 0 and 50. The stiffness level was evaluated using questions 6–7, with a total possible pain level score ranging from 0 to 20. Finally, the functional limitation was assessed using questions 8–22. The total possible pain level score ranged from 0 to 150. The internal consistency of the instrument was excellent, with a Cronbach's alpha coefficient of 0.983.

### 2.7 Analyses

Data were analyzed using both descriptive and inferential statistical methods with SPSS version 29 (SPSS Inc., Chicago, IL, USA). To blind for analysis, codes of the participants were used during analysis by N.P., who had never met the participants. Descriptive statistics included frequency, percentage, mean, standard deviation (SD), and range. Inferential analyses were conducted to compare variables between the intervention and control groups using Pearson's *χ* ^2^ test, Fisher's exact test, and independent samples *t*‐tests. The primary outcomes included scores of VAS and WOMAC. Sub‐group analysis was done for the WOMAC. Additionally, paired *t*‐tests were performed to compare pre‐ and post‐intervention VAS and WOMAC absolute scores within both the intervention and control groups. Mean differences (changed scores from pre‐ to post‐ intervention of each participant) were also calculated. Statistical significance was set at *p* < 0.05, and 95% confidence intervals were applied for all analyses.

## 3 Results

The study involved 58 participants (*n* = 29 for each intervention and control group, response rate = 100%). All participants were Buddhists, and the mean age (SD) in years was 68.24 (4.72) and 67.86 (5.29) for the intervention and control groups, respectively. No statistically significant differences in baseline characteristics were observed between the groups (See Supporting Information S1).

The study revealed no significant differences in the absolute scores of WOMAC and VAS between the intervention and control groups. However, the mean differences in VAS score among participants in the intervention and control groups differed significantly, reduced by 4.55 points and 3.48 points, respectively. In addition, the mean differences for the total score of WOMAC and each domain of WOMAC among participants in the intervention and control groups differed significantly (See Table 1).

**Table 1:** Comparison of the average score of the Visual Analog Scale (VAS) and Western Ontario and McMaster University (WOMAC) between the intervention and control group after the intervention program using independent *t*‐test statistics.

Variables	$\overset{®}{x}$	S.D.	*T*	*p* value
VAS absolute score				
Intervention group	1.97	1.180	0.144	0.886
Control group	1.93	0.530		
VAS mean difference score				
Intervention group	4.55	1.40	2.953	0.005*
Control group	3.48	1.35		
WOMAC absolute total score				
Intervention group	37.98	26.23	0.142	0.888
Control group	38.14	11.68		
WOMAC mean difference score				
Intervention group	110.41	28.30	3.526	0.001*
Control group	80.14	36.57		
WOMAC absolute pain domain score				
Intervention group	7.03	5.22	1.390	0.171
Control group	8.62	3.24		
WOMAC pain domain score—mean difference				
Intervention group	26.03	9.79	3.985	<0.001*
Control group	18.10	8.29		
WOMAC absolute stiffness domain score				
Intervention group	1.79	1.72	0.394	0.695
Control group	1.96	1.61		
WOMAC stiffness domain score—mean difference				
Intervention group	9.24	3.04	3.875	<0.001*
Control group	5.41	4.36		
WOMAC absolute functional limitation domain score				
Intervention group	28.55	20.26	0.245	0.808
Control group	27.55	8.45		
WOMAC functional limitation domain score—mean difference				
Intervention group	75.14	20.31	3.091	0.003*
Control group	56.62	25.07		

*Note:* Mean difference was calculated by pre‐intervention score minus post‐intervention score of each participant.

Abbreviation: SD, standard deviation.

*p value < 0.05.

## 4 Discussion

This study evaluated the effectiveness of the PIKOR program in reducing pain and improving knee osteoarthritis outcomes among older adults in a northern province of Thailand. The findings demonstrated that the post‐intervention absolute scores did not significantly differ between groups, participants in the intervention group demonstrated significantly greater reductions (change scores) in VAS, WOMAC total score, pain, stiffness, and functional limitation than those in the control group. The absolute total WOMAC, absolute WOMAC‐pain domain score, absolute WOMAC‐stiffness domain score, and absolute WOMAC‐functional limitation score among the intervention and control groups did not differ. The mean score of the total WOMAC, WOMAC‐pain domain, WOMAC‐stiffness domain, and WOMAC‐functional limitation among the intervention group after the PIKOR program was higher than in the control group.

Thai Traditional Massage, known in Thai as “Nuad Thai”, is Thailand's traditional manual therapy rooted in the principles of Thai traditional medicine. It covers examination, diagnosis, and treatment (non‐pharmacological treatment) [15]. CTTM, HHC, KPH, and LKS have shown promise in this context and are increasingly studied for their clinical benefits.

CTTM is a clinical therapeutic form of Thai Traditional Massage that emphasizes targeted pressure application along energy lines and trigger points to relieve musculoskeletal symptoms. It improves circulation, reduces tissue hardness, enhances heart rate variability, and modulates pain through neurological pathways such as the gate control theory [16, 17]. Unlike general Thai massage, which focuses on relaxation, CTTM is structured for rehabilitative purposes [17]. In addition, the effectiveness of CTTM includes increased skin temperature and arterial blood flow [18]. Studies support its analgesic efficacy across several conditions, including chronic tension‐type headaches [19], rheumatoid arthritis [20], and neuropathic pain [16]. As a result, the CTTM serves as a viable alternative to pharmacological analgesics for musculoskeletal disorders [16, 17, 18].

HHC, also known as “Luk Pra Kob,” involves applying a steamed muslin‐wrapped compress filled with herbs such as *Zingiber montanum* and turmeric. The compress's moist heat and herbal volatiles penetrate the skin, exerting analgesic, anti‐inflammatory, and muscle‐relaxant effects [15, 21]. Clinical trials have demonstrated the efficacy of HHC in reducing pain levels in KOA, with impact comparable or superior to topical diclofenac and oral ibuprofen [21, 22]. A meta‐analysis [23] confirmed that HHC's pain‐relieving effects were similar to NSAIDs and exercise therapies. Therefore, HHC is a culturally relevant non‐pharmacological intervention for managing musculoskeletal pain.

KPH involves the topical application of herbal paste, often containing *Zingiber montanum* and other anti‐inflammatory herbs, directly onto the knee. The poultice may be classified as “hot” (for muscle relaxation) or “cold” (for inflammation reduction) and is applied for 15–20 min per session [24]. It could demonstrate significant reductions in WOMAC and VAS scores after just three treatments [25], while being more effective in reducing pain than diclofenac gel, and without the adverse side effects commonly associated with NSAID use [26]. LKS focuses on improving knee mobility, strength, and pain reduction through structured stretching routines [27]. Recognized as a core element of musculoskeletal rehabilitation, stretching is often incorporated into multimodal programs [28]. Evidence shows that stretching alone can significantly reduce pain and improve gait and joint range of motion [29]. It also enhances the flexibility of the hamstrings in older adults, which is especially beneficial for KOA management [30].

Recently, data suggested that combining traditional Thai therapies amplifies therapeutic outcomes. Integrative programs utilizing CTTM, HHC, and KPH have demonstrated significant reductions in knee pain, joint stiffness, and improved functional mobility in KOA patients [31, 32]. Furthermore, a notable decrease in pain recurrence was observed (26.7%), indicating potential long‐term benefits [32]. While conventional treatments like diclofenac and ibuprofen remain standard for KOA pain relief, traditional Thai therapies offer a comparable, if not superior, alternative, particularly for patients with contraindications to NSAIDs. CTTM and HHC have both demonstrated clinical effectiveness similar to that of NSAIDs without side effects [21]. KPH also outperformed topical diclofenac in pain reduction. Stretching exercises (LKS) remain essential for functional recovery [26, 27]. In this study, the PIKOR program can reduce treatment time with applied Thai medicine, but the effectiveness of the treatment (CTTM, HHC, KPH, and LKS) shows no difference from standard treatment. The PIKOR program has three treatments that take 130 min, while the standard treatment lasts 180 min, and the Thai medicine doctor must always be present with the patient. However, long‐term follow‐up and other target groups are essential to establish their role in evidence‐based clinical practice.

## 5 Strengths and Limitations

This study was guided by the conceptual framework of Thai traditional medicine and was conducted using a randomized controlled trial to ensure robust methodological validity. The sample size has been calculated, and the questionnaire is valid and reliable. However, the PIKOR program is limited because it involves a short follow‐up; a longer follow‐up, such as 1–3 months, is recommended. In addition, the Consolidated Standards of Reporting Trials (CONSORT) Framework was reported [33]. The study was retrospectively registered in the Thai Clinical Trials Registry. However, it has been cross‐checked against the registry record to ensure consistency regarding study dates, intervention/control procedures, outcomes, sample size, and blinding.

## 6 Conclusions

The PIKOR program was designed to reduce pain and improve KOA among older adults and aimed to evaluate the effectiveness of the program. The PIKOR program was associated with greater reductions in pain, stiffness, and functional limitations among participants in the intervention group compared to those in the control group. Therefore, the program may serve as a potential therapeutic option for managing pain and knee osteoarthritis among older adults.

## Author Contributions

All authors contributed to the conception and design of the study. Pakorn Pusuwan, Phanit Srisuttha, Raweephun Sunanta, and Keng Chaichana were responsible for data collection. Nitikorn Phoosuwan conducted the data analysis and data visualization. All authors contributed to the drafting and revision of the manuscript and approved the final version for publication.

## Ethics Statement

The study was approved by the Ethics Committee in Thailand (COA: UP‐HEC 1.3/040/65). Permission to conduct the study was obtained from the Phayao Provincial Public Health Office and the respective District Public Health Offices. No authors were involved in delivering the treatment interventions to participants. Participant confidentiality was maintained, and privacy was strictly protected throughout the study.

## Consent

All participants were provided with both verbal and written information prior to providing written informed consent. They were provided with comprehensive information about the disease, treatment options, risks, and alternatives, as well as their right to participate in the study. Participants were also informed of their right to withdraw from the study at any time without consequence. They had an opportunity to ask any questions until they fully understood. They gave their consent voluntarily and were not coerced, intimidated, or unduly influenced in their decision‐making by signing a patient consent form.

## Conflicts of Interest

The authors declare no conflicts of interest.

## Supporting information


Supporting File

## Data Availability

The data that support the findings of this study are available from the corresponding author upon reasonable request.
