# Supplementary material for: Effectiveness of a Program for Pain Intensity and Knee Osteoarthritis Reduction Among Elderly Individuals in Thailand: A Randomized Controlled Trial Study
Source: Immun Inflamm Dis. 2026 Nov 13;14(11):e70604. doi: 10.1002/iid3.70604 (PMC13760728; doi:10.1002/iid3.70604)
Supplement: Supplementary file 1 [file IID3-14-e70604-s001.docx]

Characteristics of the participants (n = 58)

| Characteristics | Intervention group (n=29) | Control group (n=29) | p-value |
| --- | --- | --- | --- |
| Age (years) | | | |
| Mean (SD)  Range | 68.24 (4.72)  60–77 | 67.86 (5.29)  61–77 | .774^a^ |
| Gender, n (%) | | | |
| Male | 9 (31.0) | 8 (27.6) | .773^b^ |
| Female | 20 (69.0) | 21 (72.4) |  |
| Marital status, n (%) | | | |
| Married | 16 (55.2) | 18 (62.1) | .594^b^ |
| Not married (widowed/divorced) | 13 (44.8) | 11 (37.9) |  |
| Education level, n (%) | | | |
| No education | 3 (10.3) | 2 (6.9) | .99^c^ |
| Primary School or higher | 26 (89.7) | 27 (93.1) |  |
| Occupation, n (%) | | | |
| Agriculturist | 8 (27.6) | 10 (34.5) | .79^b^ |
| Employment (daily/self/government) | 9 (31.0) | 7 (24.1) |  |
| No employment | 12 (41.4) | 12 (41.4) |  |
| Body mass index (kg/m^2^) |  |  |  |
| Normal weight | 15 (51.7) | 19 (65.5) | .286^b^ |
| Overweight/obese | 14 (48.3) | 10 (34.5) |  |
| Having underlying disease(s) |  |  |  |
| Yes (hypertension/diabetes/heart/kidney) | 18 (62.1) | 21 (72.4) | .401^b^ |
| No | 11 (37.9) | 8 (27.6) |  |
| Regular exercise, n (%) | | | |
| Less than 90 min/week | 26 (69.0) | 12 (41.4) | .071^b^ |
| 91–150 min/week | 6 (20.7) | 8 (27.6) |  |
| More than 150 min/week | 3 (10.3) | 9 (31.0) |  |
| Smoking, n (%) | | | |
| Never | 21 (72.4) | 20 (69.0) | .836^b^ |
| Current/Ex-smoker | 8 (27.5) | 9 (31.0) |  |
| Alcohol, n (%) | | | |
| Never | 14 (48.3) | 16 (55.2) | .766^b^ |
| Quit alcohol | 9 (31.0) | 9 (31.0) |  |
| Currently consuming alcohol | 6 (20.7) | 4 (13.8) |  |
| Visual Analog Scale |  |  |  |
| Mean (SD)  Range | 6.52 (0.99)  5-8 | 5.41 (1.21)  3-7 | N/A |
| WOMAC scale |  |  |  |
| Mean (SD)  Range | 147.79 (18.46)  118-189 | 118.27 (35.92)  45-172 | N/A |

^a^based on independent T-test; ^b^based on Chi-square test; ^c^based on Fischer’s exact test; SD, Standard deviation; N/A, not applicable; *statistical significance at 0.05 level
